# Supplementary material for: Evaluating increases in sensitivity from NORDIC for diverse fMRI acquisition strategies
Source: Neuroimage. Author manuscript; Available in PMC 2023 Jun 1. (PMC10234612; doi:10.1016/j.neuroimage.2023.119949)
Supplement: 1 [file NIHMS1883359-supplement-1.docx]

# Supplemental Material


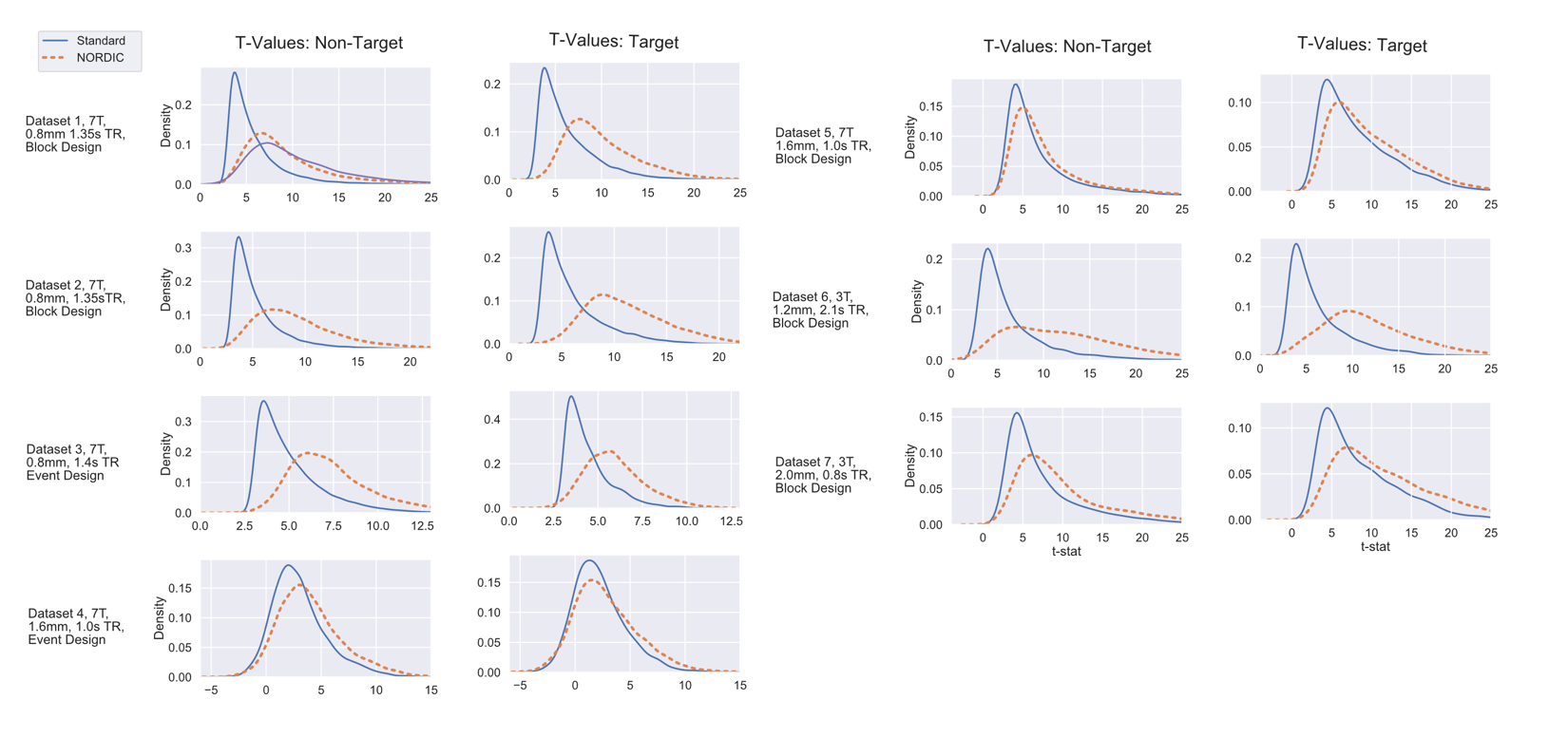
Figure S1. T-statistic histograms comparing Standard and NORDIC data within the Non-Target and Target ROIs. The distributions of t-Statistics from the model using all runs of data are shown. Distributions show the t-statistics of voxels from Non-Target (left) and Target (right) ROIs, defined as those that displayed significant positive stimulus-evoked changes relative to baseline (Non-Target) or in the contrast between Target and Non-Target conditions (Target) in the Standard data.


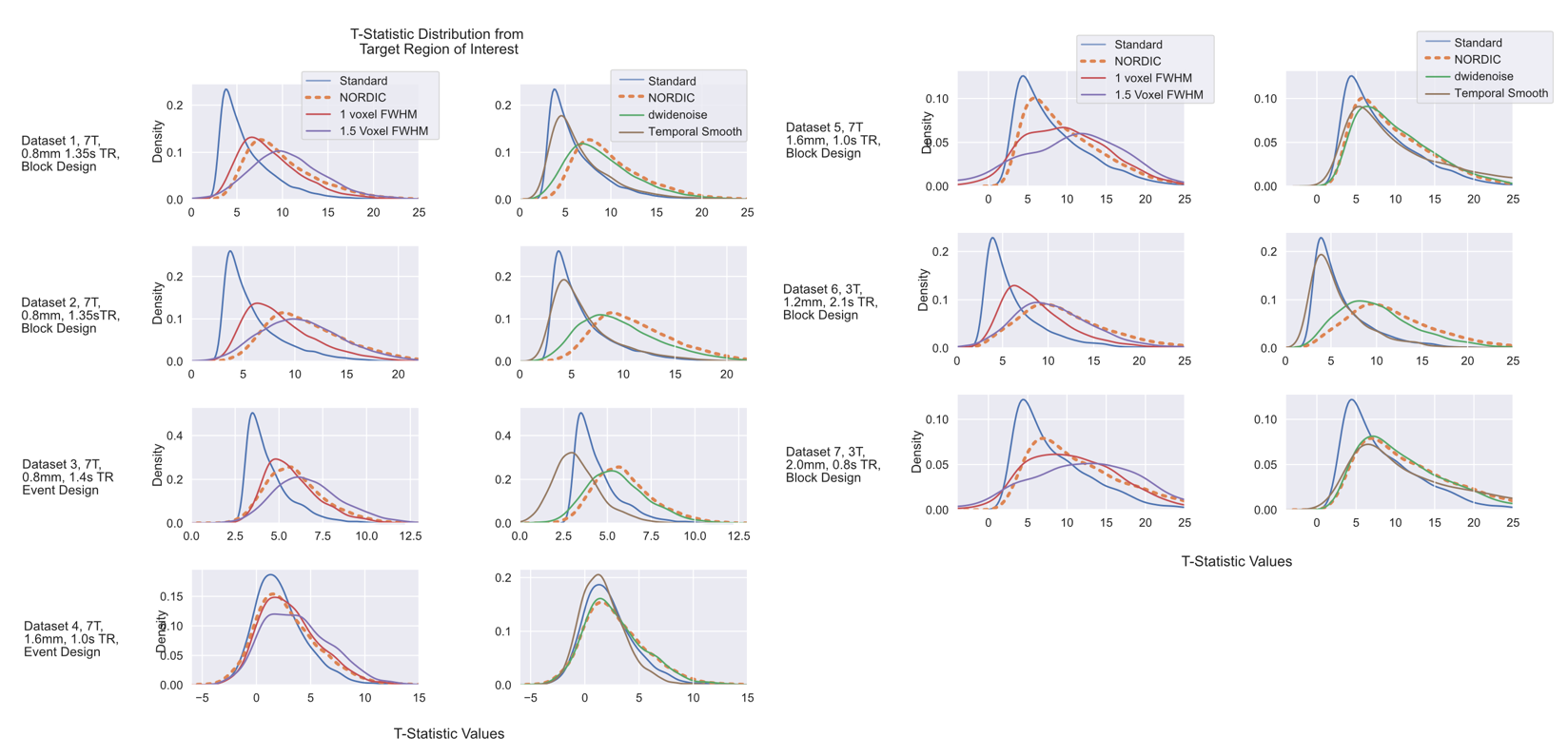


Figure S2. T-statistic values from Datasets 1 through 7 in the Target ROI, under different processing schemes within the Target ROI. Left column shows data from Standard, NORDIC and spatial smoothing with 1 and 1.5 voxel FWHM spatial smoothing. Right column compares the same Standard and NORDIC data against temporal smoothing and dwidenoise denoising. T-values were extracted from the Target ROI defined using the Standard data. The t-values obtained with NORDIC (Orange, dashed) processed data is comparable to the effects of an additional 1 or 1.5 voxels FWHM gaussian smoothing.


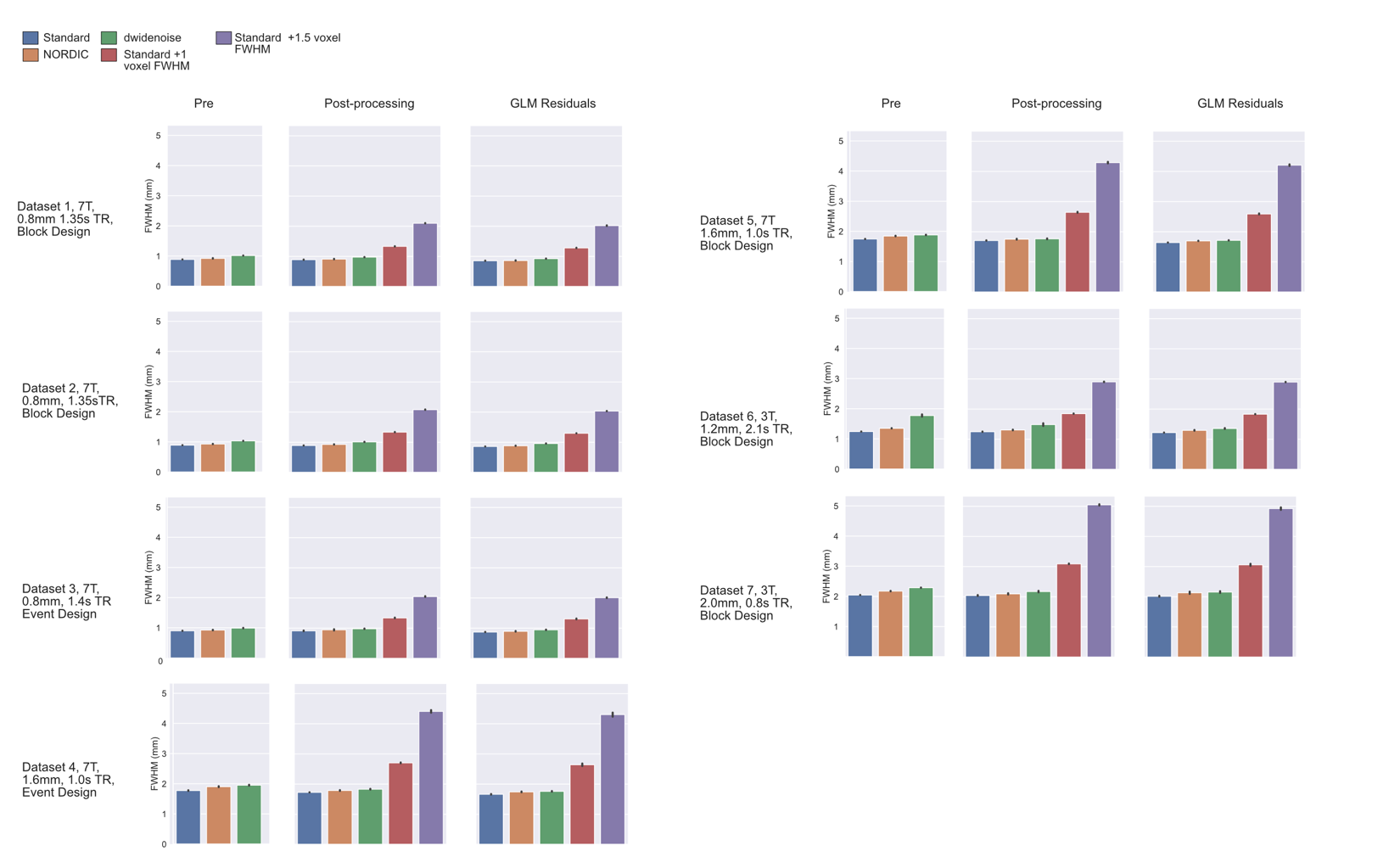


Figure S3. Global Smoothness Estimates for Datasets 1 – 7. Estimated spatial smoothness in mm (FWHM) at various processing stages for each method. Note that the image smoothness of the Standard, NORDIC, and dwidenoise data are substantially below the level of the additional 1 or 1.5 voxels of additional smoothing. These trends remain the same for the residuals (last columns) after the conventional GLM. Error bars indicate standard deviation over runs.


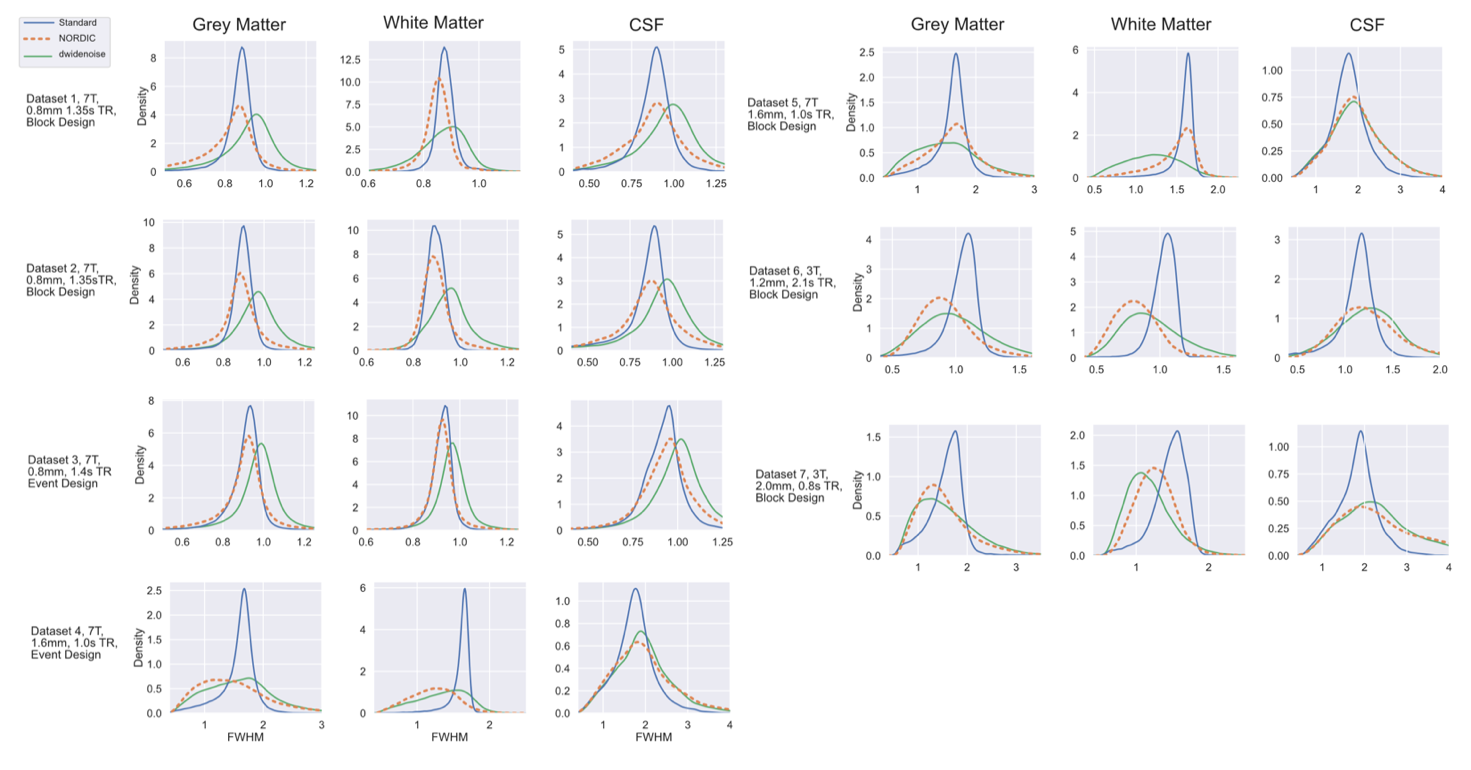


Supplemental Figure S4. Local Smoothness from Datasets 1 – 7. These kernel density estimates show the distributions of the local spatial smoothness estimates in tissue classes derived from a T1-weighted anatomical image for Standard (blue), NORDIC (orange) and dwidenoise (green).


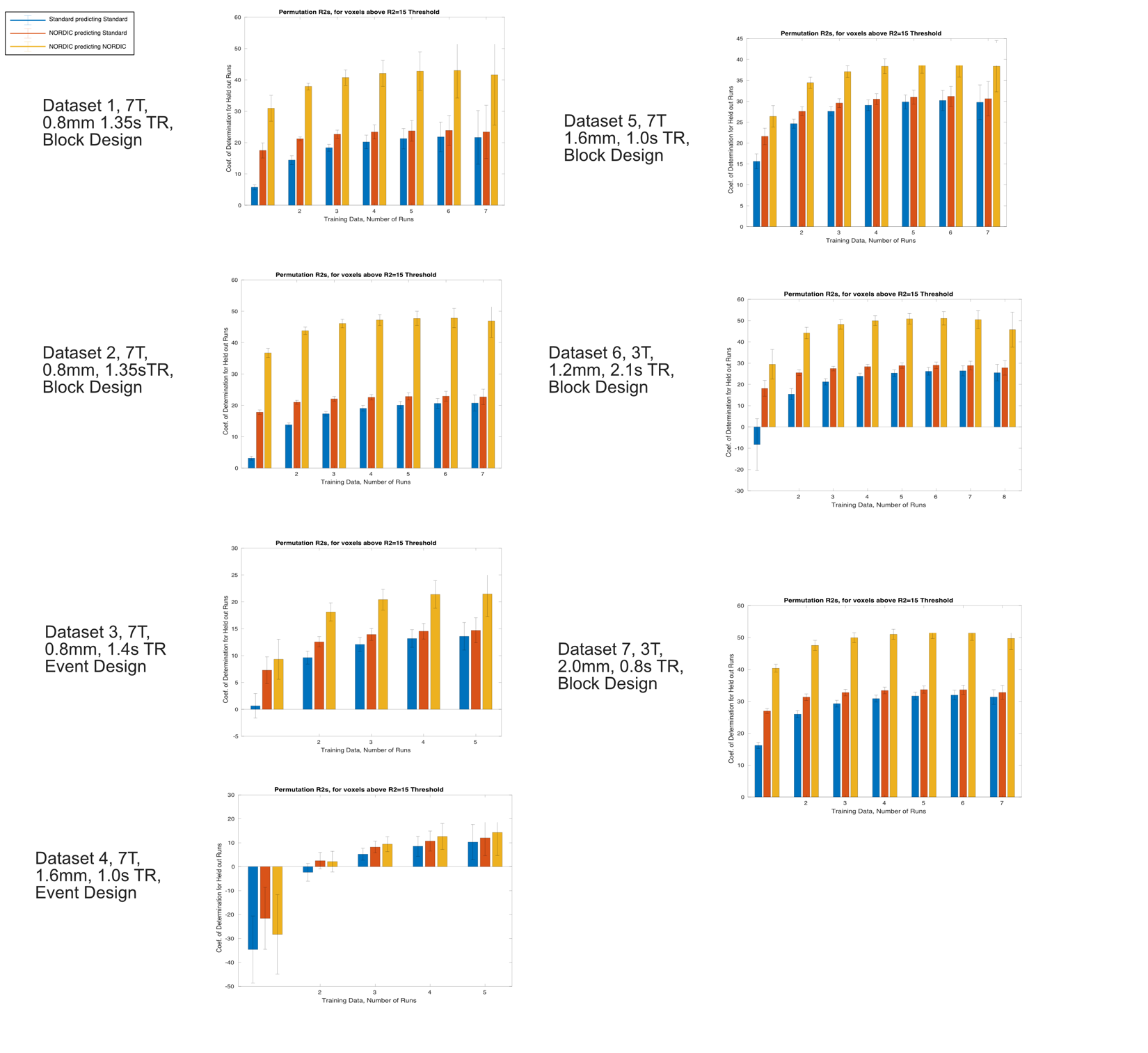


**Supplemental Figure S5. Cross Validated R^2^ for Datasets 1 through 7** Leave-p-Out training was repeated for all vales of *p* greater than 1 and less than the number of runs. The number of runs included in the training vary across the X-axis, with bar height reflecting the R^2^ obtained. Including more data allows Standard models to approach, but not reach 2 to 3 runs of NORDIC data. Error bars indicate standard deviation.

| Standard & NORDIC | Standard & Spatial Smoothing | Standard & Temporal Smoothing |
| --- | --- | --- |
| 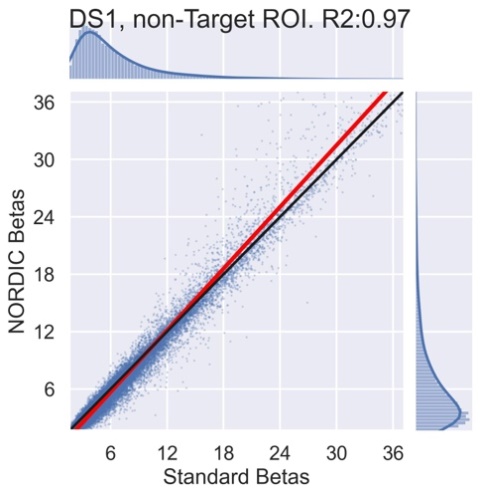 | 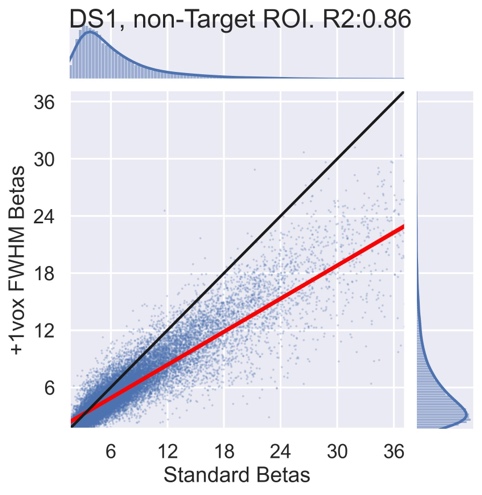 | 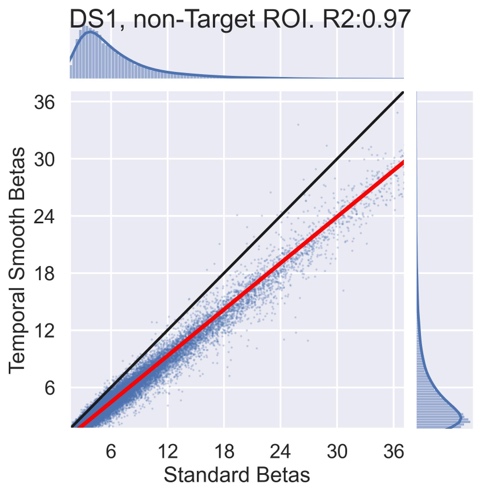 |
| 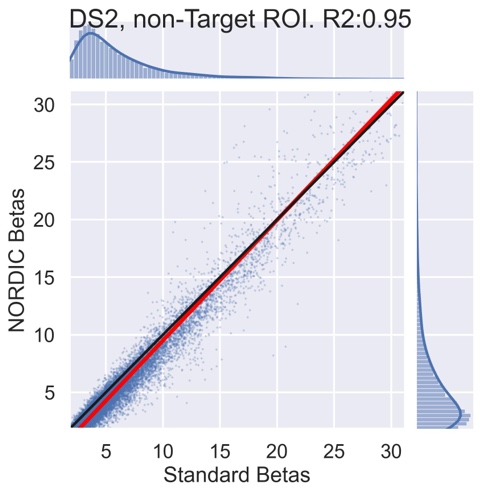 | 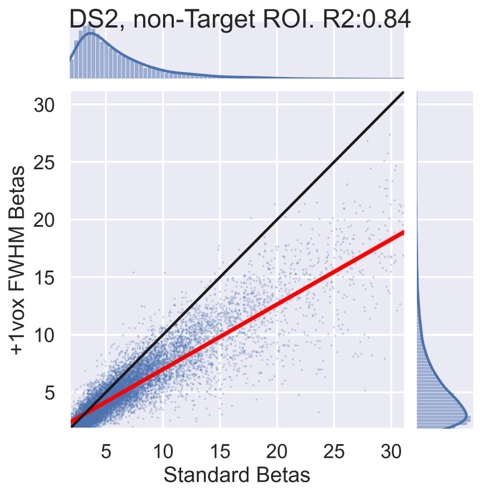 | 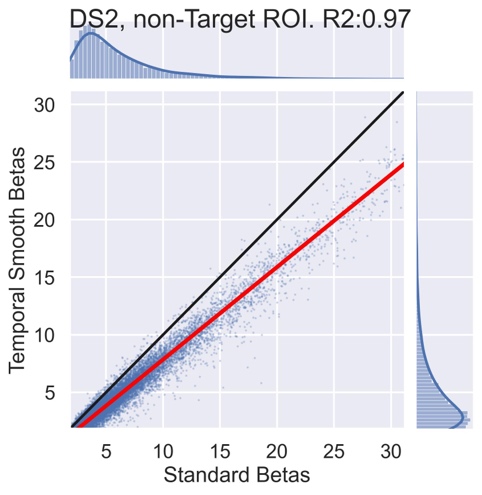 |
| 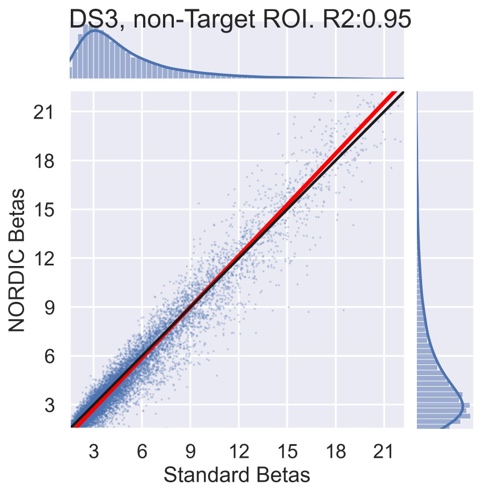 | 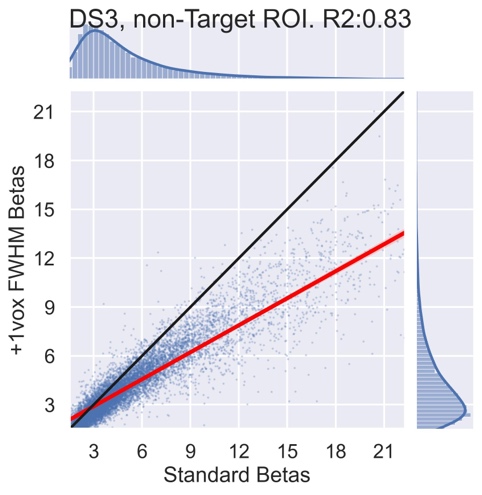 | 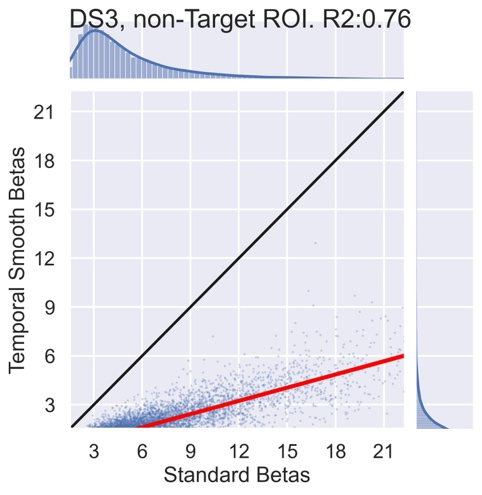 |
| 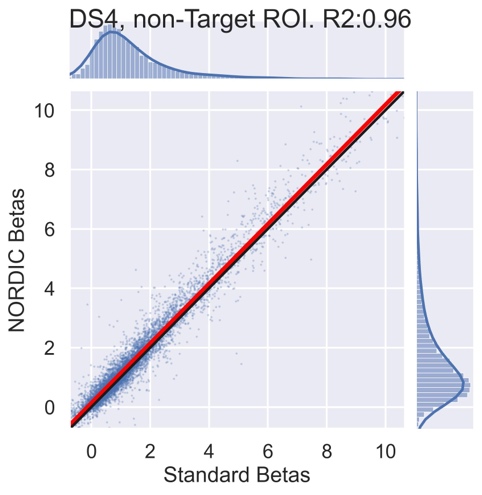 | 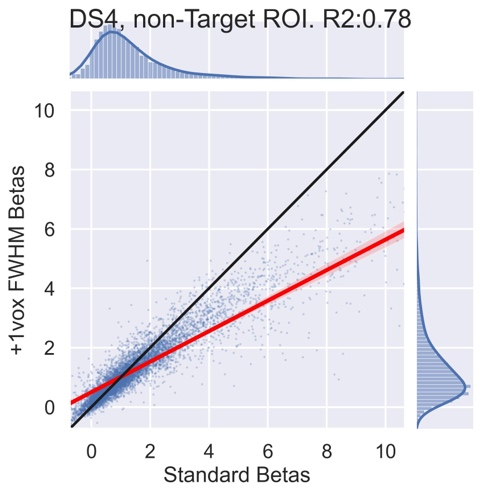 | 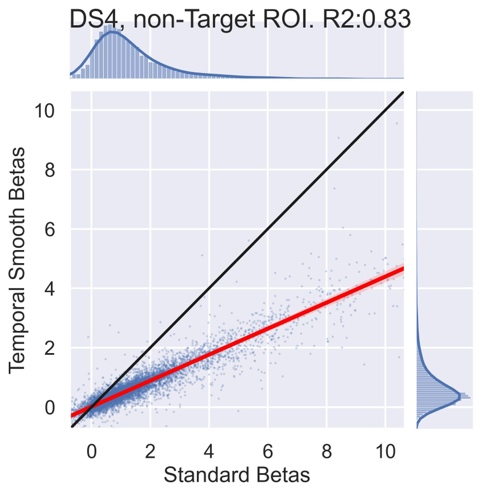 |
| 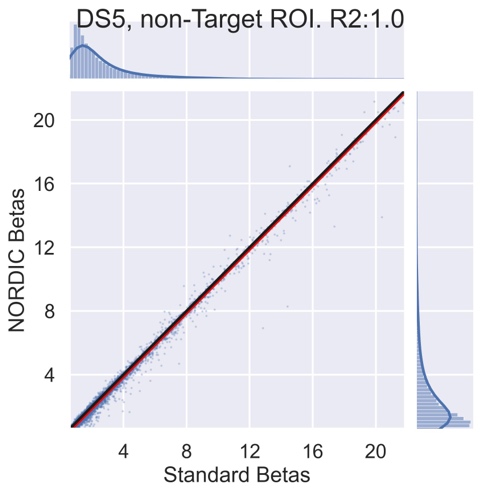 | 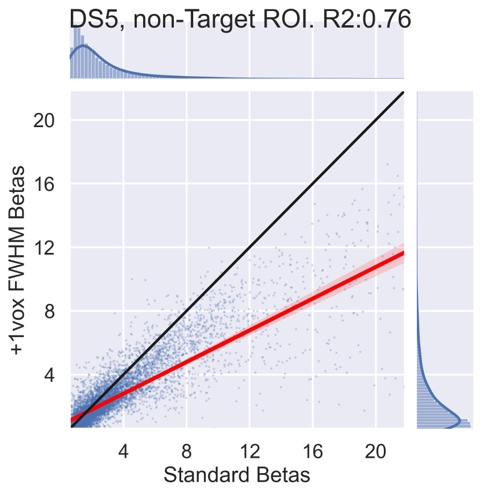 | 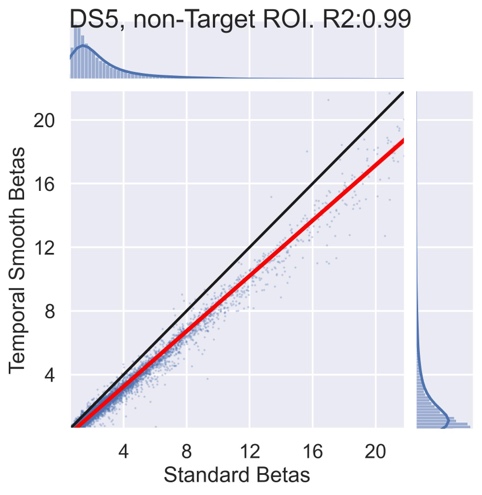 |
| 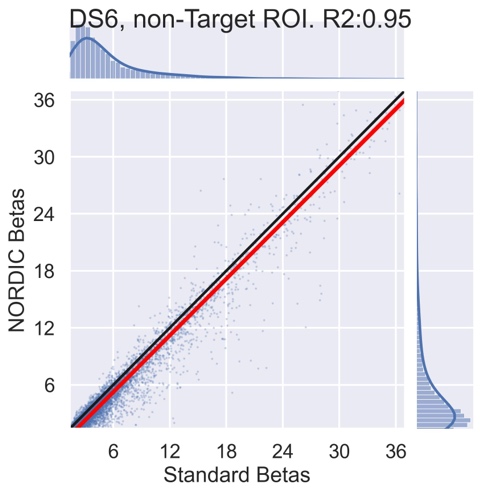 | 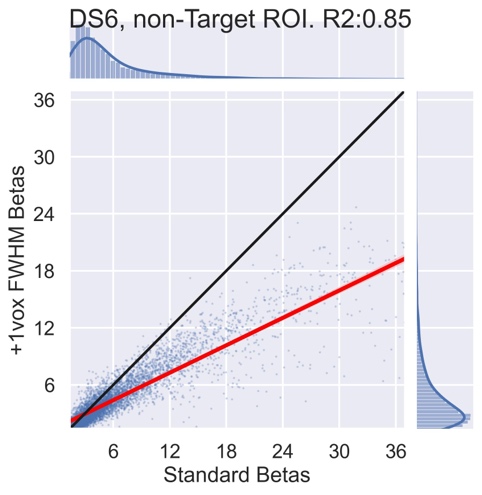 | 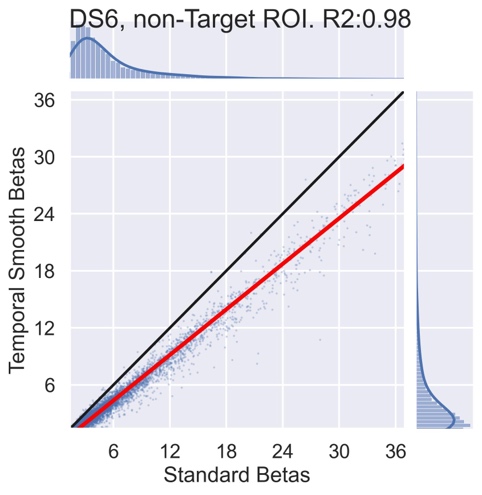 |
| 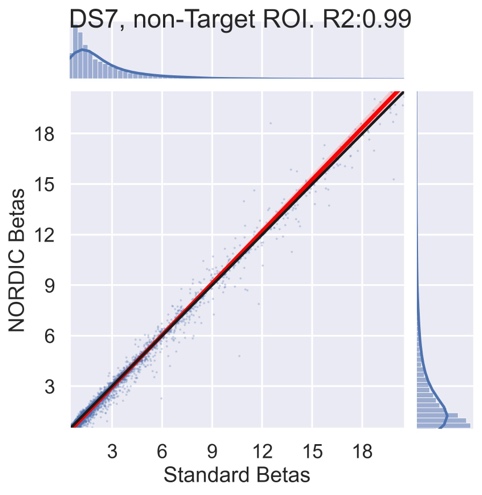 | 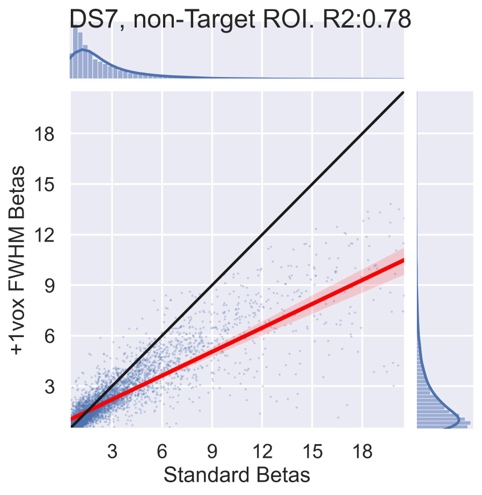 | 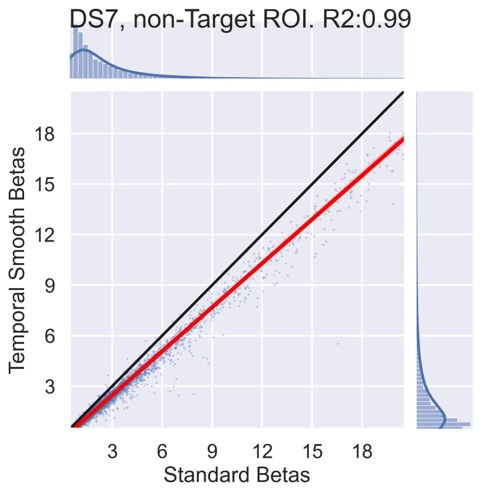 |

Supplemental Figure S6. Scatter plots showing the relationship between the activation amplitude (i.e. beta, in percent signal change) within the large Non-Target ROI for the Standard data and NORDIC (1^st^ column), +1 Voxel spatial smoothing (2^nd^ column) or temporal smoothing (3^rd^ column). The black line is unity. The red line shows a regression line fit to the points. Distributions for each datatype are shown above and to the right to highlight that the vast majority of activation amplitudes are concentrated in the lower left-hand corner of the plot. The coefficient of determination, R^2^, is provided in each plot’s title.

**Local Perturbation Response analysis**

Following the reviewers suggestion we implement the local perturbation response (LPR) method for evaluating non-linear reconstructions from Chan, C.C. and Haldar, J.P., 2021.

We tested the LPR technique on in-vivo data, and also used it on a numerical simulation with random matrices. In LPR, a small amplitude perturbation (here, a checkerboard) is added to a single time-point, and for a measurement model, **Y**, the difference NORDIC (**Y**+LPR) - NORDIC (**Y**), in reconstruction is evaluated for the ability to **recover** the injected LPR signal and the effect of **spreading** of the injected LPR signal at other time-points.

In NORDIC the effect of the LPR can be tested on the hard threshold part for each patch by considering a model Y=X+N. If the model **X**, has a low-rank representation, then the LPR, which is simultaneously a low-rank and a sparse signal, is not necessarily aligned with the subspace containing **X**. Thus, intuitively only its projection onto this subspace can be recovered. It should be noted that if the additional LPR signal is expected to represent what is observed in fMRI data, its recovery may be tackled with robust PCA, designed for a low-rank + sparse model (Candès et al., 2011), but which has additional parameters as compared with hard thresholding. However, in our experience, we do not expect such vastly different patterns to be present for a single time-frame and vanish subsequently.

For the numerical simulation for Y=X+LPR +N, LPR was selected as a 36x36 checkerboard with 6x6 squares, both X and N had dimensions 1296 x 100, to maintain a ratio of 11:1. The entries of both X and N were i.i.d. and real valued distributed with variance 1.3, and 1 respectively, and $\mathbf{X}_{\mathbf{L}}=\mathbf{u}_{X}\mathbf{s}_{X_{L}}\mathbf{v}_{X}^{T}$ was the *R*-dimensional low-rank representation of $\mathbf{X=u}_{X}\mathbf{s}_{X}\mathbf{v}_{X}^{T}$, such that for n≤R the n^th^ singular-value $\mathbf{s}_{X_{L}}^{n}=\mathbf{s}_{X}^{n}$and for n>R, $\mathbf{s}_{X_{L}}^{n}=0$. For the simulation, both the case of low-rank and full-rank model were evaluated with both separated and overlapping spectrum of singular values for the model and the added noise. As a quantitative metric for assessing the combination of noise and signal in time-points not probed by the LPR, the ratio $\epsilon={|\left| \cdot| \right|_{2, t\neq t_{0}}}/{|\left| \cdot| \right|_{2, t=t_{0}}}$was used. The four cases of LPR recovery and spreading are shown in figure S7, along with their $\epsilon$ value. For these cases, when X is low-rank the residual from LPR is more noticeable than when X is full-rank.


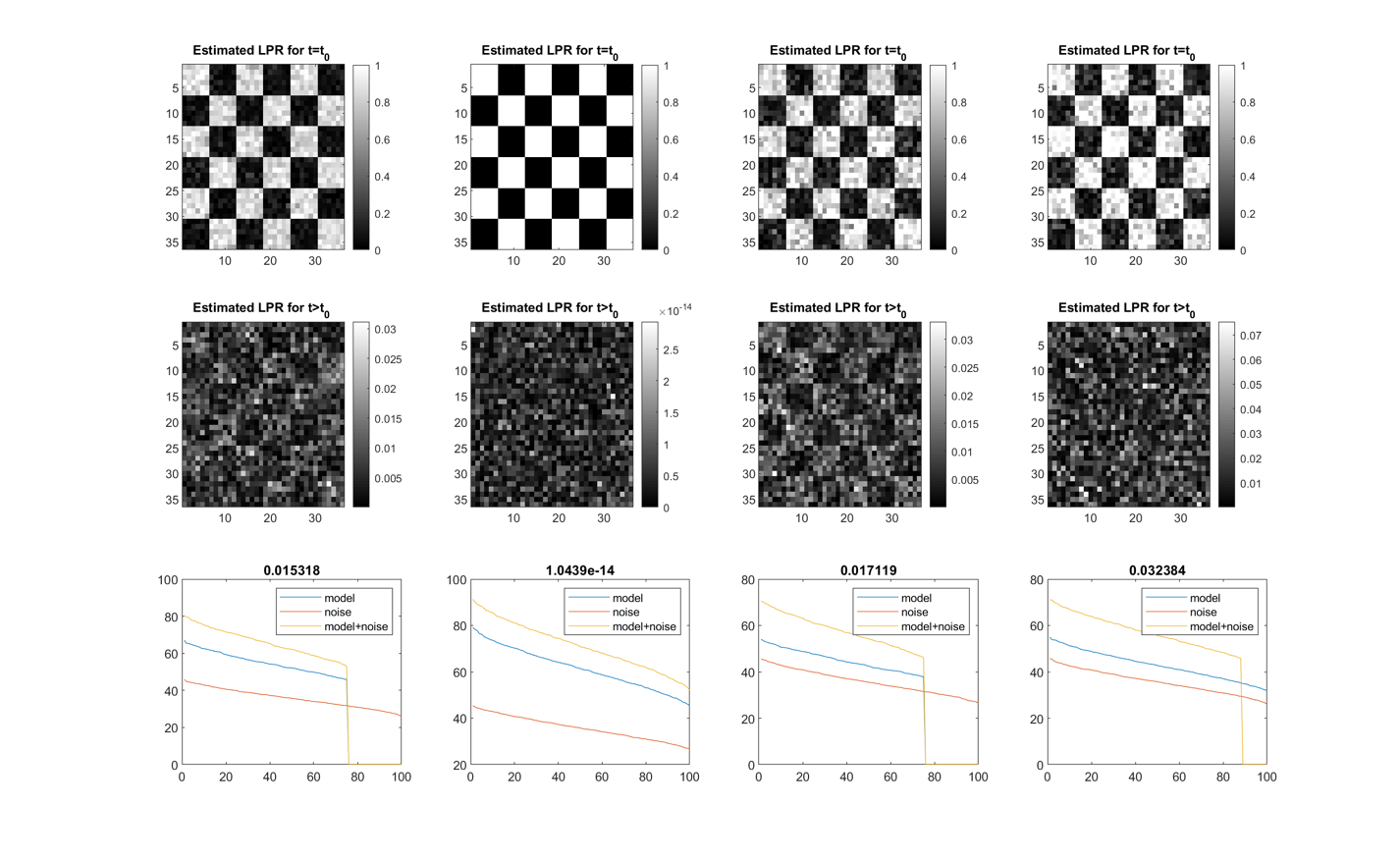


Figure S7. Numerical simulation. Four difference cases for utilizing an LPR (max(LPR)=σ)) are illustrated, for two models (low rank and full rank) and for two different noise-levels (overlapping and separated spectra of singular values).

We next added the LPR, at varying magnitudes relative to the measured thermal noise level, onto in vivo data, and performed NORDIC denoising. We then subtracted the original NORDIC data from the LPR+NORDIC data to examine to what extent the injected signal could be recovered following NORDIC.

When we consider all LPR intensities, we observe that the checkerboard LPR has decreasing signal recovery when it was of decreasing intensity relative to the thermal noise level of the data (Figure S8) and more accurate signal recovery for intensities above the thermal noise level. The LPR shows that inherent SNR is important even with techniques such as NORDIC with can identify signals below the thermal noise level and that residual noise in NORDIC is dependent on the estimated signal.


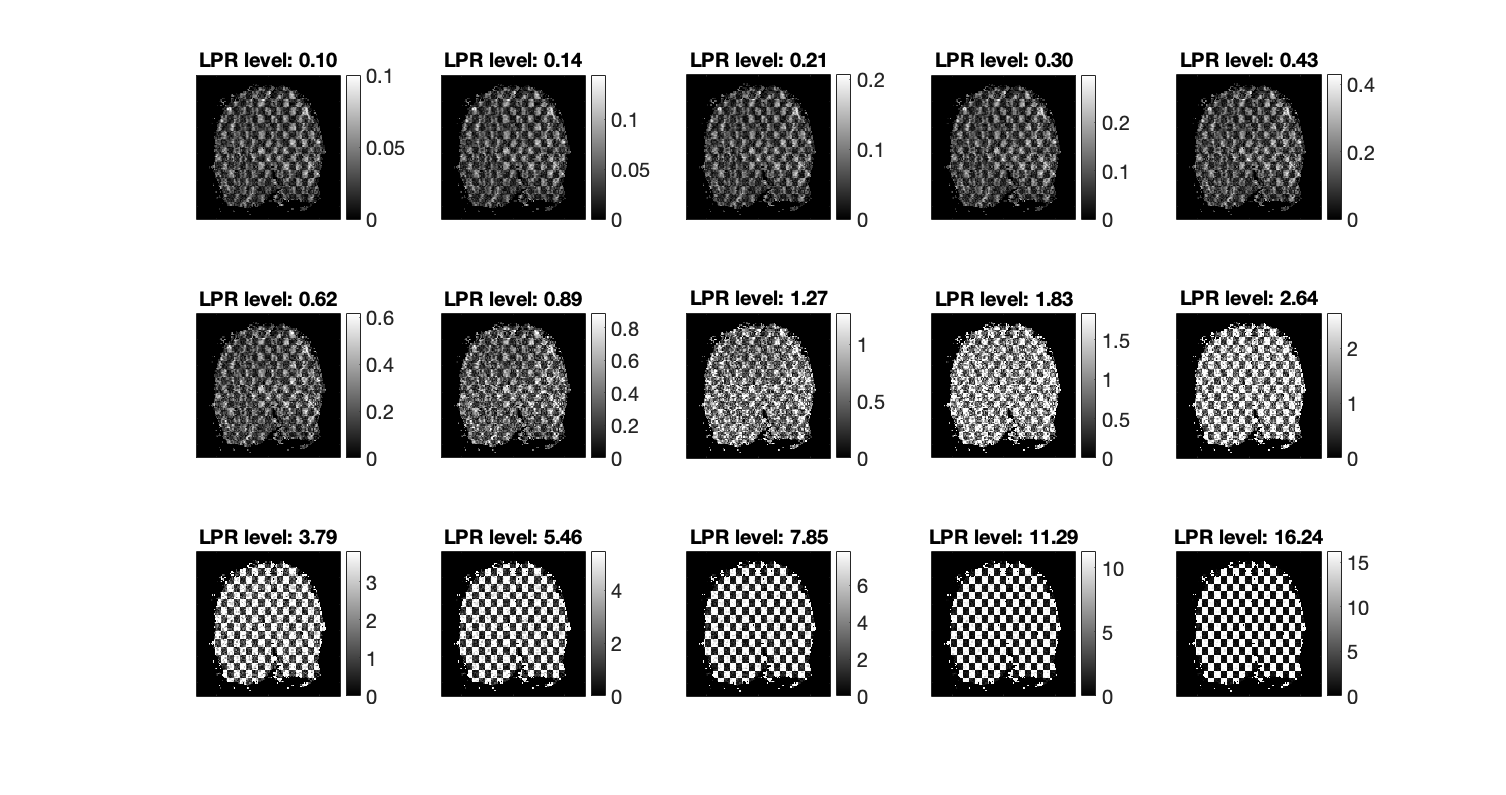


Figure S8. In vivo, recovery of injected LPR for different SNR levels. We observe that the LPR (a checkerboard) can be recovered even when the original LPR was very low in magnitude, though it is degraded.

For a neighboring timepoint (Figure S9), we find that there is very limited artifactual signal from the injected LPR, with the highest relative energy at the lower LPR magnitudes. While there is some artifact just visible, the level of this artifact is on average an order of magnitude lower than the original thermal noise or the fMRI signal fluctuations of interest. We do note that, as a proportion of the original injected LPR this artifact reaches a maximum here of 18% in two voxels for the lowest magnitude LPR. In other words, 18% of a signal that itself was 10% of the thermal noise level and thus represents a signal buried well below the thermal noise level. On average the neighboring timepoint artifact was 3.6% for this 10% LPR level, and 2.3% over all LPR levels.


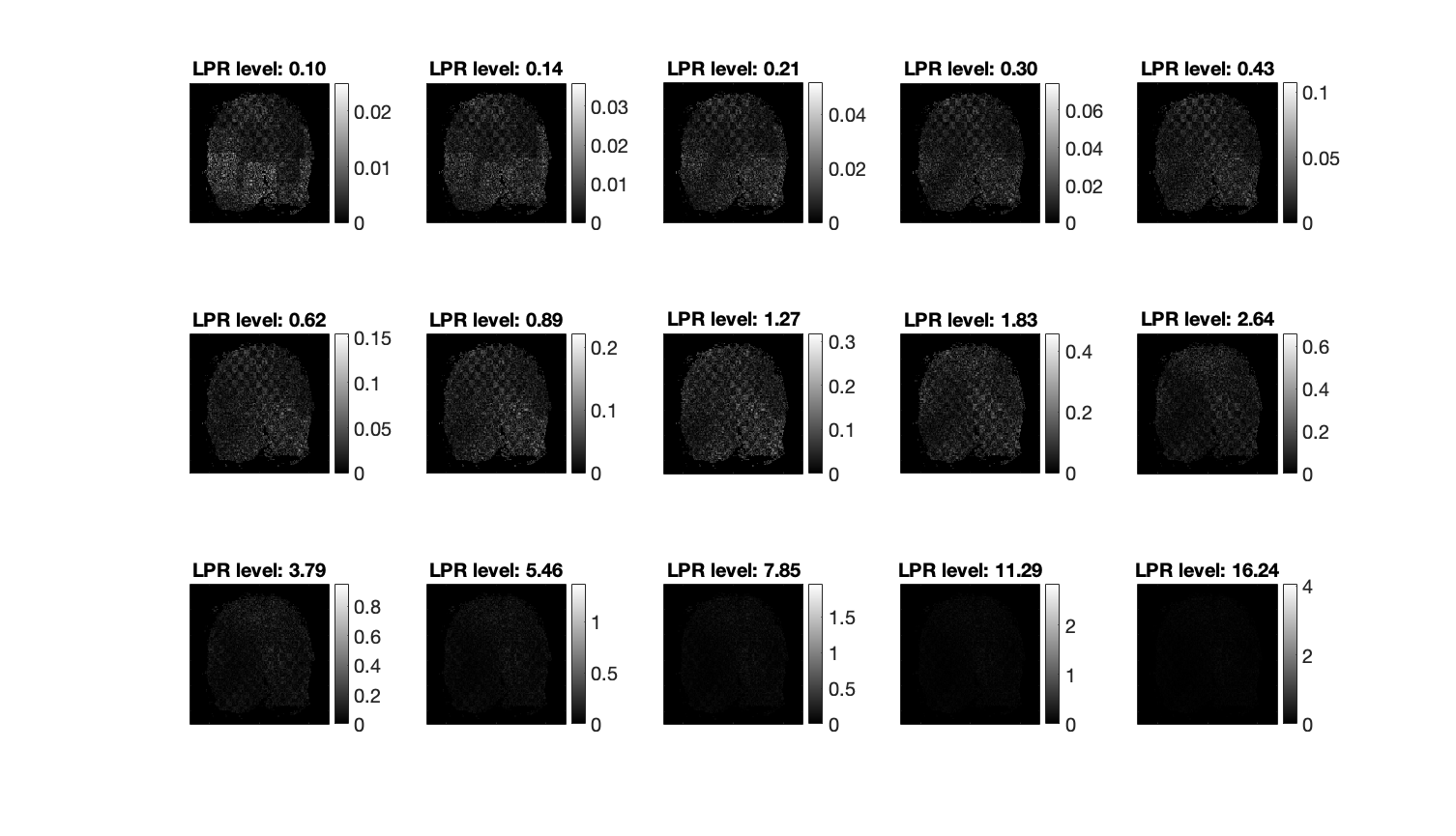


Figure S9. In vivo, comparison of neighboring timepoint after NORDIC in data with and without the LPR. The artifactual signal is at a very low intensity relative to the original injected LPR and primarily present at lower original LPR intensities (color map limits set to 25% of original LPR magnitude).

To examine the effect of this artifact, we can examine voxel time courses. Figure S10 shows the time course of 3 voxels for the original data (NO NORDIC), NORDIC and then NORDIC with 3 different LPR magnitudes. The largest effect is the suppression of thermal noise visible as the differences between the dashed black lines and the others. The effect of the spreading artifact would show up as differences between the blue lines and the 3 LPR levels – and is effectively invisible.


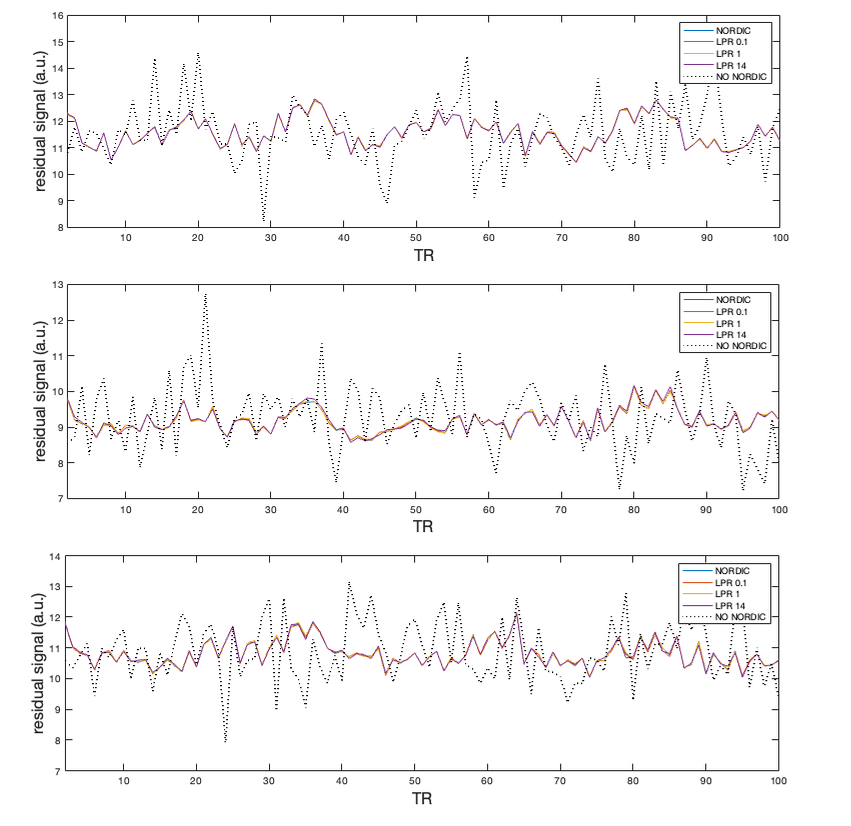


Figure S10. The minimal impact of the LPR artifact on voxel time courses. The black line shows the original data prior to NORDIC. Additional lines show the NORDIC data without the LPR (blue), and the NORDIC data with the injected LPR at various levels. While the artifact is measurable (Figures S11-S14) here we see that its effect is not meaningful. The time courses following NORDIC with and without the LPR are nearly indistinguishable.

To quantify this effect over all voxels and LPR magnitudes, we can consider the relationship between the magnitude of artifactual signal fluctuations and the magnitude of the signal fluctuations (Figure S11) following NORDIC (i.e. the temporal standard deviation). On average, this reaches a maximum of 0.02. Note that this means not that the artifact is causing 2% signal change, but rather that that the artifact is only 2% of the intrinsic fluctuations and thus very small signal changes (as visible in the voxel time courses, Figure S10).


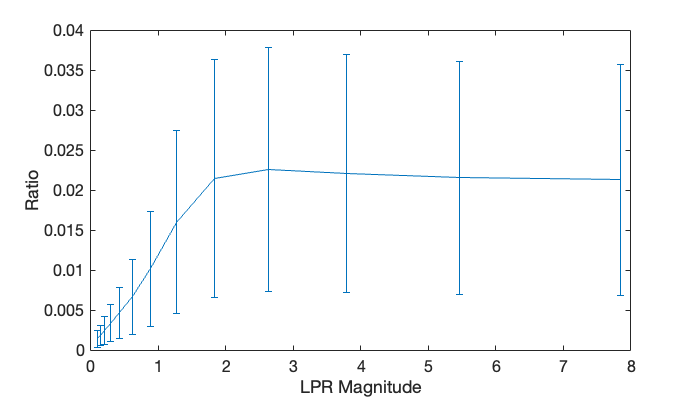


Figure S11. The magnitude of the artifactual fluctuations relative to intrinsic timeseries fluctuations. While Figures S12 and S13 showed the magnitude relative to the original LPR injection, here we are showing the magnitude of this artifact relative to the fluctuations in the denoised timeseries. While the artifact is visible (when data with and without the LPR are directly contrasted) its impact is minimal.

We can also summarize this as to the relationship of the recovered LPR and artifact signal to the original LPR signal. With an LPR for different thermal noise levels, the amount of energy recovered is close to the probed signal, and the residual is less than 1/10 of the probed signal, the plots of the simulation are shown in figure S12, with the in vivo signal shown in Figure S13.


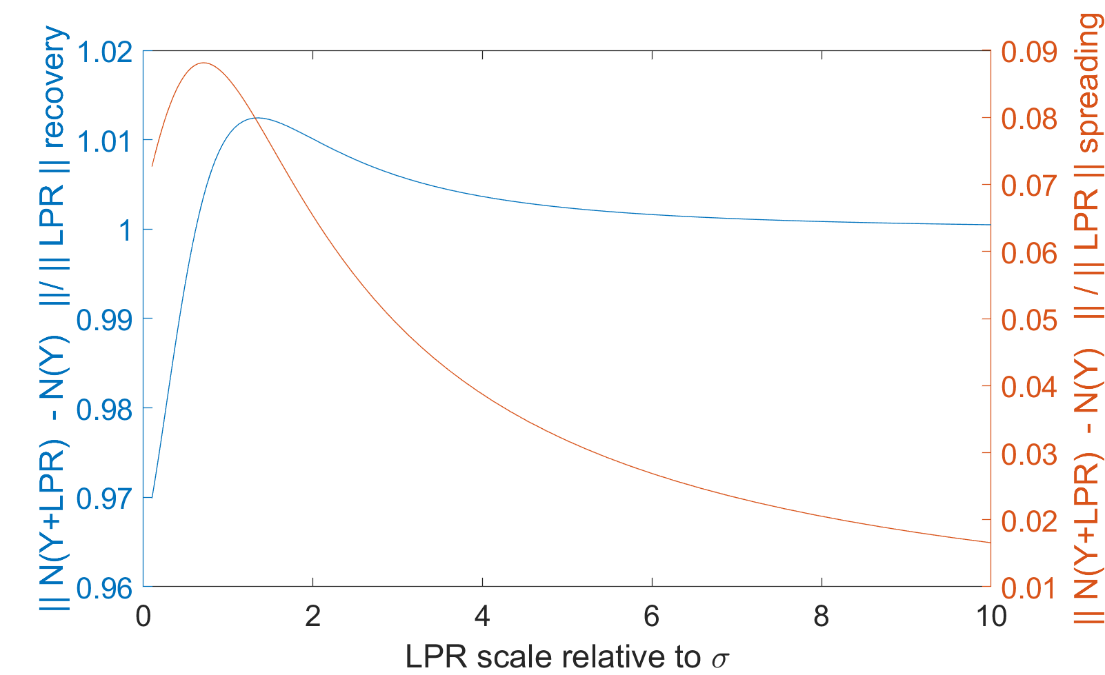


Figure S12. Plot of residual signal to LPR for different noise-regimes used in the simulation, showing recovery of LPR (blue) and LPR “artifact” at adjacent timepoints (orange).


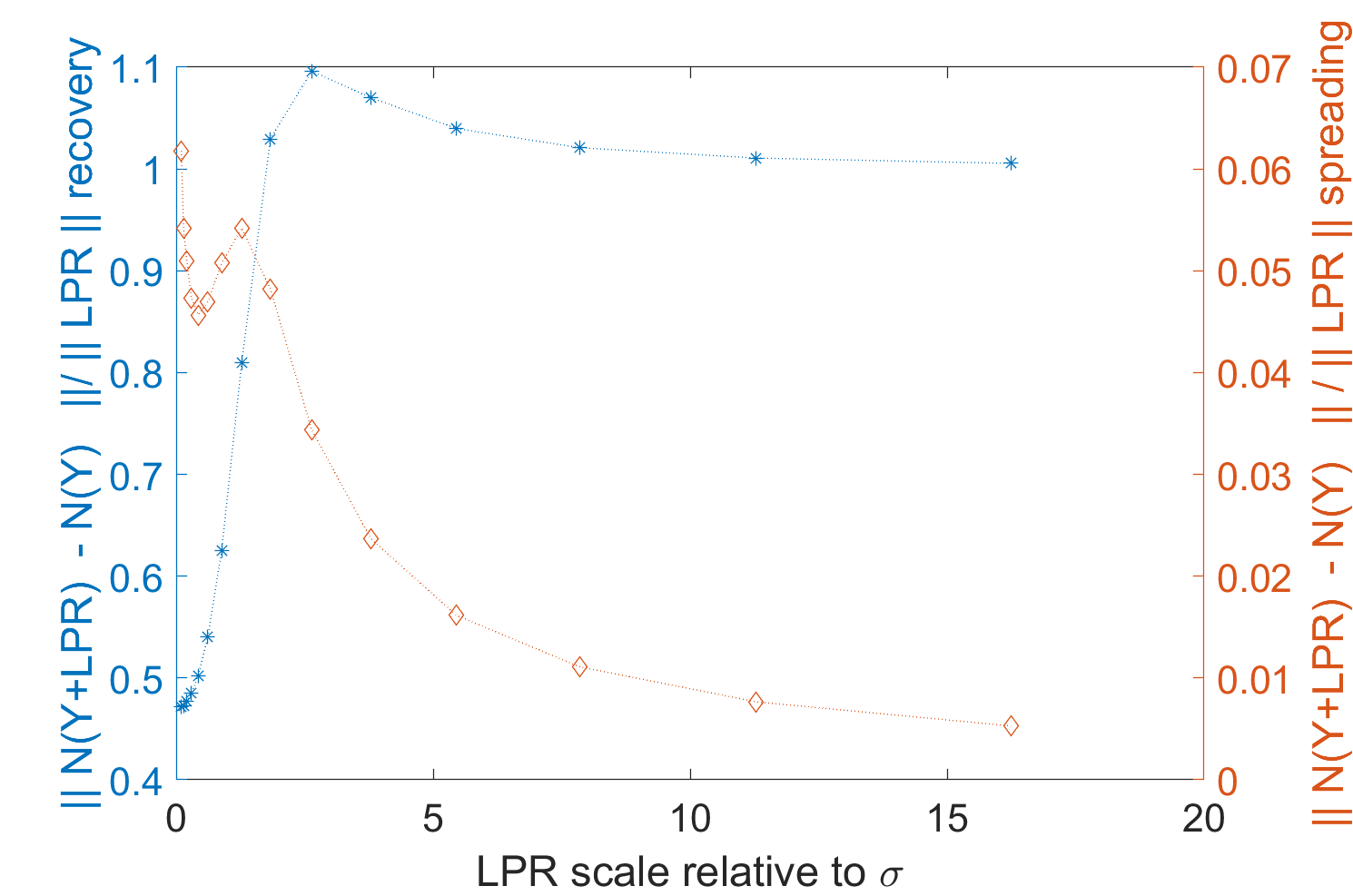


Figure S13 In vivo version of S8 showing recovery of LPR (blue) and LPR “artifact” at adjacent timepoints (orange).

From the numerical simulation the effect on the SVD of (X+LPR) vs the SVD of X for an LPR with a lower amplitude than the components in X is that the r first eigenvectors of both are almost the same, such that LPR is expressed into these basis functions, and then an r+1’th basis function is mostly identified with the remaining parts since it will be a “component”. This last basis function may or may not be recoverable, depending on the amplitude of the probed LPR. When recoverable, the representation of the LPR is the combination of the projection of the LPR onto the subspace spanned by X and any added basis function. The temporal sparse signal is likewise not described in a single explicit eigenvector but in the combination of eigenvectors. When the LPR is large, it is a large “peak” with noisy ripples for the primary eigenvector. When the LPR is low, the peak for representing a sparse signal is only achievable through the combination of eigenvectors.

A component not obtainable as being representable by $\boldsymbol{u}_{X}\boldsymbol{s}_{X}^{+}\boldsymbol{v}_{X}^{T}$ and which consistently is estimated as being (fully or partially) in $\boldsymbol{u}_{Y}\boldsymbol{s}_{Y}^{-}\boldsymbol{v}_{Y}^{T}$, will persist in the final estimation of $\boldsymbol{u}_{N}\boldsymbol{s}_{N}\boldsymbol{v}_{N}^{T}$. Such a residual component will be of less magnitude than $\boldsymbol{u}_{Y}\boldsymbol{s}_{Y}^{-}\boldsymbol{v}_{Y}^{T}$ since for some patches it otherwise would be estimated as being in $\boldsymbol{u}_{Y}\boldsymbol{s}_{Y}^{+}\boldsymbol{v}_{Y}^{T}$. What is being discarded in NORDIC are those singular vector which are embedded in the distribution of the singular vectors of Gaussian noise. The associated eigenvectors are a low-rank representation of the observed full rank noise, and those eigenvectors are indiscriminately removed.

In combination the simulation and the in-vivo data shows that for probing date with a sparse signal at the noise level, using hard thresholding on the singular values for noise removal, a residual perturbation in the denoised signal at less than 1/10 the amplitude is observable, which reflects both that not all noise is removed, and that the model in NORDIC was chosen to recover low-rank signals.

**What do we mean by removing components of the timeseries which cannot be distinguished from Gaussian distributed noise?**

For the SVD in NORDIC, the decomposition of the acquired signal may be written as

$$\boldsymbol{u}_{Y}\boldsymbol{s}_{Y}\boldsymbol{v}_{Y}^{T}=\boldsymbol{Y}=\boldsymbol{X}+\boldsymbol{N=}\boldsymbol{u}_{X}\boldsymbol{s}_{X}\boldsymbol{v}_{X}^{T}+\boldsymbol{u}_{N}\boldsymbol{s}_{N}\boldsymbol{v}_{N}^{T}$$

$${\boldsymbol{u}_{Y}\boldsymbol{s}_{Y}^{+}\boldsymbol{v}_{Y}^{T}+\boldsymbol{u}}_{Y}\boldsymbol{s}_{Y}^{-}\boldsymbol{v}_{Y}^{T}=\boldsymbol{u}_{X}\boldsymbol{s}_{X}\boldsymbol{v}_{X}^{T}+\boldsymbol{u}_{N}\boldsymbol{s}_{N}\boldsymbol{v}_{N}^{T}$$

Where $\boldsymbol{u}$ and $\boldsymbol{v}$ are matrices with eigenvectors and with eigenvectors and where $\boldsymbol{s}_{X}$ and $\boldsymbol{s}_{N}$ are diagonal matrices with the singular values for the signal and noise respectively, and $\boldsymbol{s}_{N}$ may be of full rank. The decomposition $\boldsymbol{s}_{Y}=\boldsymbol{s}_{Y}^{+}+\boldsymbol{s}_{Y}^{-}$ is constructed, based on an estimate of $\boldsymbol{s}_{N}$, such that $min(\boldsymbol{s}_{Y}^{+})>max(\boldsymbol{s}_{N}$) and $max(\boldsymbol{s}_{Y}^{-})\leq max(\boldsymbol{s}_{N})$. The estimated noise with hard thresholding in NORDIC is $\boldsymbol{u}_{Y}\boldsymbol{s}_{Y}^{-}\boldsymbol{v}_{Y}^{T}$, which is an approximation of the noise $\boldsymbol{u}_{N}\boldsymbol{s}_{N}\boldsymbol{v}_{N}^{T}$, such that all the singular values in $\boldsymbol{s}_{Y}^{-}$ is less than the largest one in $\boldsymbol{s}_{N}$, hence **NORDIC discards every principal component whose energy is smaller than NORDIC's estimate of the maximum possible energy for a subspace component from pure noise.**

It may be worth noting that the hard thresholding in NORDIC is lower than the optimal hard thresholding (Gavish and Donoho, 2014) for a low-rank signal. Likewise it may be informative to note that SVD is an orthonormal basis decomposition, where the observed signal (a row in ***Y***) is typically represented by the combination of all eigenvectors in the decomposition of ***Y***, unless the decomposition happens to create an eigenvector that exactly matches such an observed signal. By extension the estimated eigenvectors for ***X*** will be impacted by the noise observed in ***Y*** and affecting the eigenvectors in the decomposition to most compactly model ***X***. In NORDIC, the basis functions which have an importance (i.e. corresponding singular value) less than what is observable from Gaussian noise is discarded.

|  |  | **Gray Matter** | **White Matter** | **CSF** |
| --- | --- | --- | --- | --- |
| **DS1** | NORDIC | 0.82±0.148 | 0.85±0.056 | 0.89±0.214 |
|  | Standard | 0.87±0.084 | 0.88±0.039 | 0.88±0.119 |
|  | dwidenoise | 0.91±0.153 | 0.87±0.094 | 0.98±0.207 |
|  |  |  |  |  |
| **DS2** | NORDIC | 0.88±0.133 | 0.90±0.082 | 0.88±0.204 |
|  | Standard | 0.89±0.074 | 0.90±0.041 | 0.84±0.151 |
|  | dwidenoise | 0.97±0.133 | 0.96±0.102 | 0.96±0.195 |
|  |  |  |  |  |
| **DS3** | NORDIC | 0.91±0.147 | 0.92±0.089 | 0.95±0.192 |
|  | Standard | 0.91±0.10 | 0.92±0.062 | 0.92±0.138 |
|  | dwidenoise | 0.98±0.15 | 0.97±0.102 | 1.02±0.19 |
|  |  |  |  |  |
| **DS4** | NORDIC | 1.53±0.657 | 1.22±0.32 | 1.96±0.749 |
|  | Standard | 1.57±0.301 | 1.59±0.159 | 1.74±0.487 |
|  | dwidenoise | 1.62±0.592 | 1.34±0.357 | 1.95±0.667 |
|  |  |  |  |  |
| **DS5** | NORDIC | 1.6±0.476 | 1.47±0.268 | 1.98±0.622 |
|  | Standard | 1.58±0.287 | 1.58±0.157 | 1.76±0.449 |
|  | dwidenoise | 1.53±0.569 | 1.21±0.336 | 1.97±0.649 |
|  |  |  |  |  |
| **DS6** | NORDIC | 0.93±0.238 | 0.81±0.191 | 1.24±0.36 |
|  | Standard | 1.04±0.154 | 1.03±0.102 | 1.13±0.202 |
|  | dwidenoise | 1.00±0.289 | 0.94±0.247 | 1.25±0.33 |
|  |  |  |  |  |
| **DS7** | NORDIC | 1.54±0.57 | 1.26±0.273 | 2.5±1.15 |
|  | Standard | 1.57±0.344 | 1.46±0.236 | 1.82±0.473 |
|  | dwidenoise | 1.58±0.6 | 1.18±0.311 | 2.36±0.929 |

Supplemental Table S1. Mean and Standard Deviation of Local Smoothness Estimates in mm FWHM for Datasets 1 through 7.

|  | **NORDIC, dwidenoise** | **Standard, dwidenoise** | **NORDIC, Standard** |
| --- | --- | --- | --- |
| **DS1** | 0.999±0.0008 | 0.999±0.0014 | 0.999±0.0012 |
| **DS2** | 0.997±0.003 | 0.992±0.0058 | 0.993±0.0054 |
| **DS3** | 0.998±0.0031 | 0.998±0.0024 | 0.997±0.0044 |
| **DS4** | 1±0.0001 | 1±0.0003 | 1±0.0003 |
| **DS5** | 1±0.0001 | 1±0.0002 | 1±0.0002 |
| **DS6** | 0.999±0.0018 | 0.993±0.0098 | 0.989±0.0173 |
| **DS7** | 1±0.0002 | 0.999±0.001 | 0.999±0.001 |

Supplemental Table 2. The average Pearson correlations between the motion correction parameter estimates, with standard deviation over independent runs.

|  | **Standard Variability** | **NORDIC Variability** | **% Reduction** |
| --- | --- | --- | --- |
| **DS1** | 122.36 | 71.96 | 41.2 |
| **DS2** | 123.56 | 63.60 | 48.5 |
| **DS3** | 48.39 | 30.15 | 37.7 |
| **DS4** | 43.58 | 30.21 | 30.7 |
| **DS5** | 65.60 | 54.68 | 16.6 |
| **DS6** | 102.41 | 59.67 | 41.7 |
| **DS7** | 103.55 | 83.07 | 19.8 |

Supplemental Table 3. A comparison of the variability of FIR estimates within the target ROI for each dataset. This was calculated as the average (over voxels within the ROI mask) sum (over the time axis of the FIR) of the voxel-wise standard deviation over runs of the FIR response curves for the main task in each dataset (e.g. The center condition for DS1).

**Supplementary References**

Candès, E.J., Li, X., Ma, Y., Wright, J., 2011. Robust principal component analysis? J. ACM 58 11:1-11:37 doi:10.1145/1970392.1970395.

Gavish, M., Donoho, D.L., 2014. The optimal hard threshold for singular values is $4/\sqrt 3$. IEEE Trans. Inf. Theory 60, 5040–5053. doi:10.1109/TIT.2014.2323359.
